# The Acids of Fruits

**Published:** 1890-06

**Authors:** 


### THE ACIDS OF FRUITS.

Mr. George W. Johnson, in his *Chemistry of the World*, says of our common table vegetables :

" The grateful acid of the rhubarb leaf arises from the malic acid and bin-oxalate of potash which it contains ; the acidity of the lemon, orange and other species of the *genus citrus* is caused by the abundance of citric acid which their juice contains ; that of the cherry, plum, apple and pear, from the malic acid in their pulp ; that of gooseberries and currants, black, red and white, from a mixture of malic and citric acids ; that of the grape from a mixture of malic and tartaric acids ; that of the mango from citric acid and a very fugitive essential oil ; that of the tamarind from a mixture of citric, malic and tartaric acids ; the flavor of asparagus from aspartic acid, found also in the root of the marsh-mallow ; and that of the cucumber from a peculiar poisonous ingredient called fungin, which is found in all fungi, and is the cause of the cucumber being offensive to some stomachs. It will be observed that rhubarb is the only fruit which contains bin-oxalate of potash in conjunction with an acid. It is this ingredient which renders this fruit so wholesome at the early commencement of the summer, and this is one of the wise provisions of nature for supplying a blood purifier at a time when it is likely to be most needed. Beet root owes its nutritious quality to about nine per cent. of sugar which it contains, and its flavor to a peculiar substance containing nitrogen mixed with pectic acid. The carrot owes its fattening powers also to the sugar, and its flavor to a peculiar fatty oil ; the horse radish derives its flavor and blistering power from a volatile acrid oil ; garlic and the rest of the onion family, derive their peculiar odor from a yellowish, volatile acrid oil ; but they are nutritious from containing nearly half their weight of gummy and glutinous substances not yet clearly defined."

